# Tele-medicine versus face-to-face consultation in Endocrine Outpatients Clinic during COVID-19 outbreak: a single-center experience during the lockdown period

**DOI:** 10.1007/s40618-020-01476-2

**Published:** 2020-12-23

**Authors:** F. Ceccato, G. Voltan, C. Sabbadin, V. Camozzi, I. Merante Boschin, C. Mian, V. Zanotto, D. Donato, G. Bordignon, A. Capizzi, G. Carretta, C. Scaroni

**Affiliations:** 1 Endocrinology Unit, Department of Medicine DIMED, University-Hospital of Padova, European Reference Network On Rare Endocrine Conditions (endoERN) Center of Padova, Via Ospedale Civile, 105, 35128 Padova, Italy; 2 Department of Neuroscience DNS, University of Padova, Padova, Italy; 3 Department of Surgical Oncological and Gastroenterological Sciences (DiSCOG), University of Padova, Padova, Italy; 4 Department of Directional Hospital Management, University-Hospital of Padova, European Reference Network On Rare Endocrine Conditions (endoERN) Center of Padova, Padova, Italy

**Keywords:** Endocrine unit, COVID-19, Telemedicine, Hub-spoke organization

## Abstract

**Context:**

The COVID-19 outbreak in Italy is the major concern of Public Health in 2020: measures of containment were progressively expanded, limiting Outpatients’ visit.

**Objective:**

We have developed and applied an emergency plan, tailored for Outpatients with endocrine diseases.

**Design:**

Cross-sectional study from March to May 2020.

**Setting:**

Referral University-Hospital center.

**Patients:**

1262 patients in 8 weeks.

**Interventions:**

The emergency plan is based upon the endocrine triage, the stay-safe procedures and the tele-Endo. During endocrine triage every patient was contacted by phone to assess health status and define if the visit will be performed face-to-face (F2F) or by tele-Medicine (tele-Endo). In case of F2F, targeted stay-safe procedures have been adopted. Tele-Endo, performed by phone and email, is dedicated to COVID-19-infected patients, to elderly or frail people, or to those with a stable disease.

**Main outcome measure:**

To assess efficacy of the emergency plan to continue the follow-up of Outpatients.

**Results:**

The number of visits cancelled after endocrine triage (9%) is lower than that cancelled independently by the patients (37%, *p* < 0.001); the latter reduced from 47 to 19% during the weeks of lockdown (*p* = 0.032). 86% of patients contacted by endocrine-triage received a clinical response (F2F and tele-Endo visits). F2F visit was offered especially to young patients; tele-Endo was applied to 63% of geriatric patients (*p* < 0.001), visits’ outcome was similar between young and aged patients.

**Conclusions:**

The emergency plan respects the WHO recommendations to limit viral spread and is useful to continue follow-up for outpatients with endocrine diseases.

## Introduction

In early 2020, the world experienced the Severe Acute Respiratory Syndrome Coronavirus (SARS-CoV-2) infection, rapidly labelled as global pandemic by the World Health Organization (WHO) [1]. The clinical spectrum of Coronavirus Disease 2019 (COVID 19) ranges from asymptomatic carriers [2], to patients with mild/self-limiting respiratory tract illness, up to those with severe progressive pneumonia [3], multi-organ failure, and death [4, 5].

People affected by different endocrine diseases are facing their own disease and the risk of COVID-19 infection; however, the clinical impact of the SARS-CoV-2 in this subset of patients is not already kwon [6–10]. COVID-19 treatment might induce endocrine complications, as adrenal insufficiency secondary to the high-dose glucocorticoid treatment, which is sometimes used in patients with severe disease [11, 12]. SARS-CoV-2-related subacute thyroiditis may be an underestimated manifestation of COVID-19 [13], and adrenal infarction can represent a factor of poorer prognosis [14]. Recently, dexamethasone has been reported effective to reduce 28-day mortality among patients receiving invasive mechanical ventilation or oxygen at randomization [15]. A direct effect on hypothalamic–pituitary–adrenal axis or thyroid cells was described during previous SARS outbreak in 2002–2003 [16, 17]. Despite the aforementioned acute presentation, most of the common endocrine diseases (thyroid or adrenal autoimmunity, secondary hypertension or osteoporosis, pituitary/thyroid/adrenal tumours) are long-term conditions; therefore, increased awareness, motivation about diagnostic path and self-management are of paramount importance in affected patients [18–20].

First cases in Italy and in Veneto (one of the most SARS-CoV-2-affected area) were reported since the end of February [21]. Further clusters were identified, and measures of containment were progressively expanded, enforcing social distancing, isolation and quarantine of all positive cases and their relatives. Despite such procedures, at the time of this submission (October, 2020), COVID-19 achieved more or less 450,000 infections and 37,000 deaths in Italy (respectively, 38.000 and 2.300 in Veneto, data from the Italian National Institute of Health [22]). Moreover, Italy is approaching the second outbreak wave, as most of others European countries. The Italian Government superimposed the lockdown in March 2020: a redefinition of the rules generally applied to the Italian National Health-care System (NHS) forced us to change the organization of our Endocrine Outpatients clinic.

An implementation-oriented resilient NHS is characterized by the capability to develop new models/policies in case of unexpected event [23], as SARS-CoV-2 outbreak. Therefore, we had not only to face the COVID-19 infection, but also to change our usual approach at the Outpatient Clinic, from the traditional face-to-face (F2F) visit to new models, as tele-Medicine consultation: it can allow clinicians to maintain the doctor–patient relationship.

We have quickly developed a care pathway tailored for the Endocrine Outpatients Clinic in a biohazard scenario. We have collected the first results of its application during the weeks of lockdown to assess its adequacy.

## Materials and methods

### The endocrine triage

On March 8th, 2020, the Italian Government announced a new decree imposing the quarantine of 13 different municipalities in Northern Italy. Few days later (March, 11th), lockdown policies were applied to Italy. Regarding outpatients, only first visits suggested by the Emergency Department or by the General Practitioners (GPs) were allowed with F2F modality. First or follow-up visits dedicated to pregnancy or to patients with a neoplasm (thyroid or adrenal cancer, pituitary adenoma, neuro-endocrine tumours, parathyroid disease and their follow-up) could be performed, if social distancing was guaranteed.

Health-care providers (HCPs) in the Veneto Region are organized as a Hub-Spoke model [24]. University-Hospital (AOU, Azienda Ospedale-Università) of Padova is one of the European Reference Network for Rare Endocrine Disease (Endo-ERN) Center. Endocrine Outpatient Clinic covers all fields of endocrinology, from the first level as a spoke for the inhabitants of Padova, to the most complex cases requiring dedicated expertise and multi-disciplinary evaluation, as a hub centre for Italian patients. In our Endocrine Unit, first-level endocrinology (the spoke) is organized in 15 dedicated rooms every week, dedicated to all new referrals (new patients) or to the follow-up of thyroid autoimmune disease or nodules, metabolic diseases, gynaecological endocrinology and non-fractured osteoporosis. GPs, other Physicians or emergency department suggest patients’ first-access. Follow-up visits are planned during the previous assessment. We have a dedicated Endocrine Service for pregnant women, with thyroid ultrasound (US) facility. Second-level Endocrinology (the hub) is divided in Neuroendocrinology, Adrenal Disease, Thyroid Disease, Gynaecological Endocrinology, Rare Diseases, Autoimmune Endocrinology, Osteoporosis and PTH disease.

Medical history is always available: clinical data are reported in the web-based database of the AOU Padova, used as an electronic Case Report/Record Form (eCRF).

**Fig. 1:**
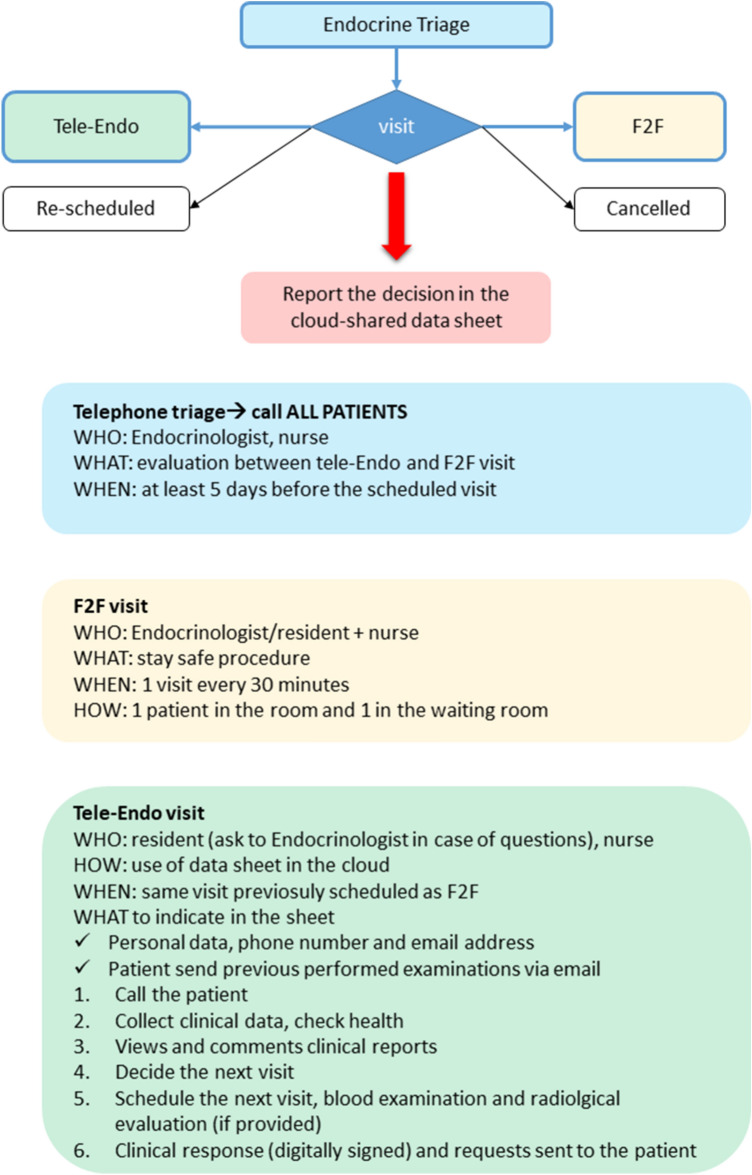
The “endocrine triage”

All visits must be scheduled by reservation: during COVID-19 outbreak, the reorganization of the outpatient clinic was necessary. To ensure the health of patients and health workers (HWs), we have developed the endocrine triage, reported in Fig. 1. The endocrine triage is a proactive measure, performed by phone and based upon a specific template that assisted the endocrinologist in making the decision between tele-Endo and F2F visit.

After the endocrine triage, the decision between F2F or tele-Medicine visit was recorded in an electronic sheet, shared in each room and available only for registered physicians/nurses. A dedicated phone number has been established, operated by a Physician and active h8.00–h18.00. Some of Endocrine HWs (nurses, residents or physicians) were temporarily relocated in the COVID-19 wards.

In case of F2F visit, the stay-safe procedures indicated in Table 1 have been adopted to reduce the risk of COVID-19 infection.

**Table 1:** The “Stay-safe” procedures

Action	Explanation
Dedicated signage	To ensure that patients understand the fixed route, the social distancing, the hands cleaning with dedicate gel and the appropriate use of masks
Social distancing	At least 1 m, with dedicated place marked on the floor for chairs, desks and waiting people
Visit duration	From 20–30 min, to ensure sufficient time for the stay-safe procedures
Nurse at entrance gate	Checks body temperature in all patient with a Non-Contact Handheld Cutaneous Infra-Red Thermometer for Fever Screening (avoid entrance if > 37.5°), gives instructions for the path, use of hydroalcoholyc gel, rational use of Face Mask (entry to rooms is prohibited in the absence of it)
Definition of a fixed route	All patients and caregivers (provided only for minors, elderly or disabled people) must enter from one door ⟶ they are accepted (by a nurse) ⟶ rest in the waiting-room (one per room), then they enter into the Endocrine Room when the previous patient has left it
Room quick sanitization	Cleaning the desk, computer screen and keyboard, examination bed with dedicated disinfectants (after every patient)

Thyroid US evaluations and fine needle aspiration (FNA) were subjected to a triage: those dedicated to known or suspected thyroid cancer were performed during lockdown weeks. An increased protection (in addition to the stay-safe procedures) was applied to the physician during the US/FNA procedure.

### Tele-medicine applied to endocrinology: tele-Endo

To guide the Endocrinologist during the endocrine triage, a multiple-choice questionnaire (with yes/no answer) has been adopted. After phone-agreement with the patient, tele-Endo was proposed in case of at least one positive item among:

- Patient confined at home for COVID-19 infection (after hospitalization, asymptomatic or with mild symptoms) or quarantine, including their relatives.
- Follow-up visits;
- Age > 65 years;
- Clinical assessment considered by endocrinologist not absolutely necessary;
- Endocrine treatment started 6 months before;
- Recognized ability to use email (with the help of family-members/caregivers if needed)

As depicted in Fig. 1, tele-Endo is planned before the scheduled visit appointment:

1. At least 5 working days before the visit, an Endocrinologist calls the patient, checking the health status (signs or symptoms related to COVID-19 infection and/or the endocrine disease). During the phone call, endocrine disease’s related blood and radiological examinations, requested during the previous visit, are checked.
2. The decision between F2F visit and tele-Endo is indicated to the patient.
3. If the patients refused the visit, or if examinations have not been already performed, the appointment is cancelled or re-scheduled. To ensure that patients are not lost-to-follow up, a new prescription for an Endocrine visit (in the former case) or a novel appointment (in the latter case) is provided.
4. If the patient agrees to tele-Endo, all the medical reports have to be sent to a dedicated email address (if not available in the local eCRF or in the Fascicolo Sanitario Elettronico Regionale, FSEr).
5. During tele-Endo visit, the endocrinologist asks the patient detailed information regarding health status, assesses correct assumption of drugs and if necessary suggests modifications of the schedule, views and comments clinical reports, plans next visit.
6. The visit report form (digitally signed), with the prescription of next control and examinations, is sent to the patient’s email. A digital version of the report is stored in the web-based service of the AUP of Padova, and is available for GPs in the web-based service of the Veneto region (FSEr).

Also, the communication with other primary-care centers (other spokes in Veneto) were co-ordinated by mail.

### Statistical analyses

To understand the number of visits, as well as the application of our suggested protocol in the real-life of Endocrine Service, we collected the number of visits performed during the lockdown weeks, and we compared them with the number of visits performed in the same weeks in 2019, calculating the difference (termed ∆^2019–2020^). We compared the rate and type of visits, the use of dedicated email, the adequate endocrine control (adapted to the specific disease and considered as a dichotomous variable yes/no), the modification of the schedules of endocrine treatment and the time proposed for the next visit. We divided our data according to age of presentation and first–second level (spoke/hub) endocrinology.

All patients gave their consent; the study was conducted in accordance with the principles of the Declaration of Helsinki. The Ethics Committee of AUP of Padova approved the study (number 37907-2020). Clinical data reported in eCRF were collected anonymously.

This observational study was conducted in accordance with the Strengthening the Reporting of OBservational studies in Epidemiology (STROBE) guidelines for cohort, case–control, and cross-sectional studies [25].

Proportions and rates were calculated for categorical data; continuous data were reported as means and standard error. Groups were compared by chi-square test for categorical variables and by the student’s t test for quantitative variables. The database was managed and statistical analysis performed by SPSS 24 software package for Windows (2016, IBM-SPSS, Armonk, New York, USA). Significance level was set as a *p* < 0.05 for all tests. All data analysed during this study are included the data repositories of the University of Padova (Research Data UniPD [26]).

## Results

**Fig. 2:**
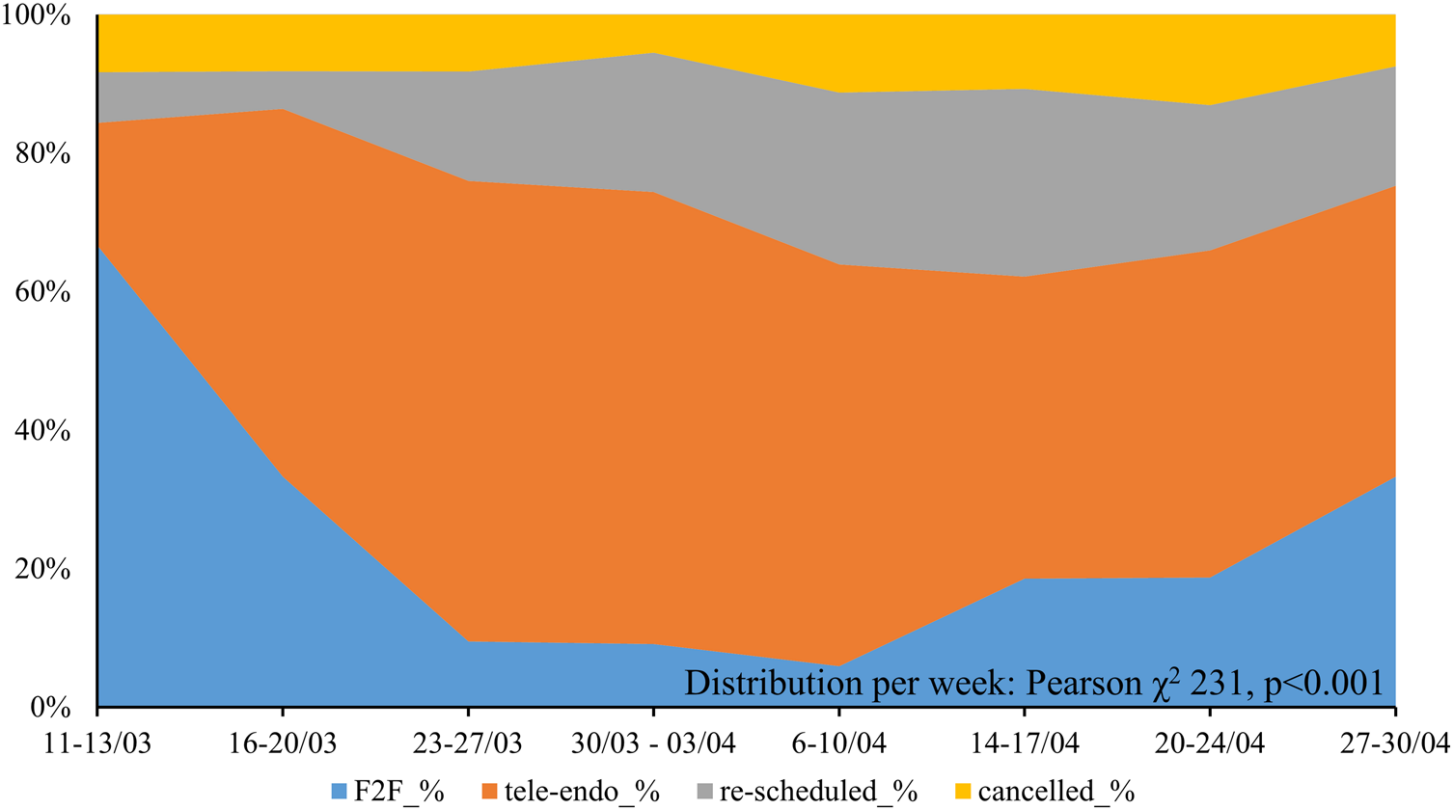
Distribution of all visits, according to the decision during the endocrine triage, in the weeks of lockdown

In Table 2, we have described the number and the type of visits performed during the weeks of lockdown, considering that the beginning of the second week of March (10th and 11th) was still characterized by normal routine activity. Considering the third week of March (from 16 to 20^th^) the actual start of the endocrine triage, we observed that the number of patients that cancelled their visit without prior consultation by the endocrine triage decreased from 47 to 19% (*y* = 0.0371*x *− 0.5231, *R*^2^ = 0.7797, *p* = 0.032, reported in Fig. 2). The number of visits cancelled after endocrine triage (9%) is far lower than the proportion of visits cancelled by patients (36.6%, *p* < 0.001).

**Table 2:** Distribution of all patients managed with the “endocrine triage” during the weeks of lockdown

Week	Total patients	F2F	Tele-endo	Total performed	Re-scheduled	Cancelled	First visits	Follow-up visits	∆^2019–2020^
March, 11–13	101	64 (66.7%)	17 (17.7%)	81 (84.4%)	11 (8.2%)	8 (8.3%)	16 (19.8%)	65 (80.2%)	− 43 (− 30.9%)
March, 16–20	155	49 (33.1%)	78 (53.1%)	127 (86.1%)	16 (6.4%)	12 (8.2%)	27 (21.3%)	100 (78.7%)	− 132 (− 47.3%)
March, 23–27	164	15 (9.2%)	105 (66.3%)	120 (75.5%)	31 (16.2%)	13 (8.1%)	19 (15.8%)	101 (84.2%)	− 121 (− 43.4%)
March, 30–April, 04	168	15 (9%)	107 (65.1%)	122 (74%)	37 (20.4%)	9 (5.5%)	18 (14.8%)	104 (85.2%)	− 115 (− 41.2%)
April, 6–10	169	10 (5.9%)	98 (58.0%)	108 (63.9%)	42 (24.9%)	19 (11.2%)	10 (9.3%)	98 (90.7%)	− 110 (− 39.4%)
April, 14–17	146	26 (18.3%)	61 (43.3%)	87 (61.6%)	44 (28%)	15 (10.7%)	13 (14.9%)	74 (85.1%)	− 75 (− 34.9%)
April, 20–24	178	33 (18.8%)	83 (47.2%)	116 (65.9%)	39 (21.0%)	23 (13.1%)	7 (6.0%)	99 (85.3%)	− 103 (− 36.9%)
April, 27–30	181	58 (33.1%)	73 (42%)	131 (75%)	37 (17.5%)	13 (7.5%)	12 (9.2%)	119 (90.8%)	− 41 (− 19.1%)
Mean	156	34 (24.4%)	78 (49.1%)	112 (73.5%)	30 (17.3%)	14 (9%)	15 (13.9%)	95 (85.1%)	− 92 (− 36.6%)

10–11/03 still normal activity at the endocrine service; 13/04: service close for Easter Monday; 01/05: service close for Labor Day. ∆^2019–2020^: difference between the considered week of lockdown and the same week in 2019

As depicted in Fig. 2, most of the patients contacted by endocrine-triage received a clinical response: the sum of visits performed combining F2F and tele-Endo modality ranged from 62 to 86% weekly. The F2F visits were strictly subjected to the “stay-safe procedures” and none of the employers in the Endocrine Outpatient Clinic (physicians, residents and nurses) have been infected. Notably, all HWs of the AOU of Padova underwent a routine molecular swab for SARS-CoV-2 every 3 weeks. Considering admission for SARS-CoV-2 infection in the AOU of Padova, three outpatients were hospitalized for COVID-19, one with pneumonia. During contact-tracing activity, none of the infection was related to the follow-up visit.

Overall, 369 patients (31.8%) contacted by the endocrine triage were ≥ 65 years of age. According to the previously reported criteria, F2F visit was offered especially to young patients, while tele-Endo was applied to 63.4% of patients with ≥ 65 years (*p* < 0.001). The number of re-scheduled or cancelled visits was reduced in geriatric patients. Use of dedicated email, adequate endocrine control and modification of the endocrine treatment were similar in the two classes of age (< or ≥ 65 years), as reported in Table 3. Moreover, the time proposed for the next visit was similar between F2F and tele-Endo visit (7.5 ± 7 vs. 8.2 ± 6.2 months, *p* = 0.605).

**Table 3:** Distribution of patients according to the age of presentation

	Age at visit < 65 years	Age at visit ≥ 65 years	*p*
F2F (*n* = 269)	210 (78.1%)	59 (21.9%)	< 0.001
Tele-endocrinology (*n* = 622)	388 (62.4%)	234 (37.6%)	
Re-scheduled (*n* = 157)	118 (75.2%)	39 (24.8%)	
Cancelled (*n* = 112)	75 (67%)	37 (33%)	
First visits (*n* = 159)	115 (72.3%)	44 (27.7%)	0.231
Follow-up visits (*n* = 1001)	676 (67.5)	325 (32.5%)	
Use of dedicated email	276/599 (46.1%)	141/292 (48.3%)	0.535
Adequate disease control	393 (66.8%)	179/289 (61.9%)	0.152
Modification of treatment	200/588 (34%)	111/288 (38.5%)	0.188

**Fig. 3:**
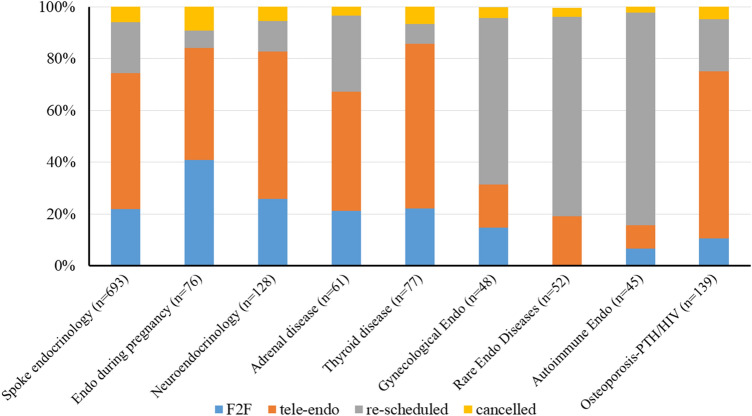
Distribution of all visits after endocrine triage, according to the different setting

In Fig. 3, we summarized the endocrine-triage applied to the second-level endocrinology. The number of performed visits (either F2F or tele-Endo) was similar according to different endocrine diseases, however, most of the patients with adrenal insufficiency were re-scheduled, because of their disease-related high-risk during COVID-19.

During the 8 weeks of lockdown, after the dedicated endocrine triage 114 thyroid ultrasound (US) evaluations have been performed (82% female, mean age 52 ± 15 years, ∆^2019–2020^ − 80%). Overall, 46 were follow-up US for differentiated thyroid cancer, 36 for thyroid nodes or multinodular goitre, 16 for new-onset hyperthyroidism. 27 FNA were performed.

## Discussion

In 2020, the COVID-19 outbreak presented a significant impact in all NHSs, because thousands of patients with severe complications were hospitalized [1, 3, 4]. On the other hand, the larger part of SARS-CoV-2-infected patients presented with mild symptoms or are asymptomatic carriers [2], increasing the risk in Outpatients Clinic. Therefore, an effort in the organization of NHSs had to be performed, in order to reduce the risk of infection for patients and HWs [7], and to continue the management of long-term chronic conditions, as endocrine diseases. Little by little that the lockdown rules were imposed in the middle of March, we have prepared in few days an emergency plan, suitable for routine clinical practice, able to respond to the health requests of patients and to minimize the spread of the infection. Notably, in this paper, we described our experience with the first application of the emergency plan, reporting the clinical activities during the lockdown weeks, based upon the endocrine triage, the stay-safe procedures and the tele-Endo consultations. Therefore, the design of this paper is not that of a trial or a case–control group.

The follow-up of patients with chronic endocrine diseases is of outmost importance to reduce/prevent comorbidities, particularly in those assuming a long-term treatment. Therefore, one of our aim was to limit the cancellation of visits. We have decided to propose a proactive approach: to call each patient before his/her access into the Endocrine Clinic, which takes place by reservation. In order to reduce the communication gap between patients and endocrinologists, we have implemented the general helpline for endocrine emergencies phone number and institutional email. The ∆^2019–2020^ is the number of patients that cancelled their visit without a previous consultation through the endocrine triage, therefore, those who did not receive an adequate counselling. In the first weeks of lockdown, the number of visits that the patients decided to cancel by themselves was high, while it progressively decreased from March to April, thanks to the application of endocrine triage model. In case of F2F visit, the stay-safe procedures have been adopted to reduce the viral spread: dedicated signage of the rooms, social distancing, dedicated nurse, temperature control and the rational use of face mask. At the end of each visit, a quick sanitization of the room was planned. Endocrine triage was useful also for first visits that have been rescheduled, because a consultation with an endocrinologist is helpful to suggest the correct time to postpone the visit, according to the disease. To conclude, one of the principal results of endocrine triage is that the number of patients’ cancellation of their visit reduced during time, according to the increased use of endocrine triage and tele-Endo. This approach is also useful to limit the number of re-scheduled visits, reducing the overload of the NHSs in the next months. In a high-risk scenario, we observed that our approach (based upon phone triage and tele-medicine) was useful to continue the follow-up of chronic patients.

Tele-Endo visit was considered after a tailored and careful selection of patients, at least 5 working days before the scheduled visit. After the patient’s agreement to the new setting of assessment, all the medical reports suggested during the previous visit were sent to the dedicated email. The tele-Endo visit was scheduled at the same time than the previous visit planned before COVID-19 outbreak. During tele-Endo, the Endocrinologist asks the patient detailed data regarding health status, checks and comments the medical reports, suggests the modification of the treatment and plans next visit. Then, the digitally signed report form with all the prescriptions is prepared and sent to the patient’s email. Three quarters of the over a thousand patients that had been contacted by the Endocrine Triage received an updated clinical response regarding their endocrine disease, considering the sum of F2F and tele-Endo visits. We paid a particular attention to those patients with adrenal insufficiency, since they are at high risk [27]. Most of them (and their relatives) are continuously educated to self-manage the risk of an adrenal crisis, by our Endocrinologists and nurses. Moreover, they can contact our Endocrine Clinic by email or phone in case of critical illness to obtain updated information regarding the management of adrenal crisis, including the modification of their glucocorticoid substitutive therapy. The endocrine triage for patients with adrenal insufficiency included also a remind of the “sick-days rules”, because initial symptoms of COVID-19 disease (fatigue, malaise, gastrointestinal symptoms, and diarrhoea) are common to those observed in patients with adrenal insufficiency [28] as a consequence of an insufficient substitutive treatment. Tele-Endo was particularly useful in elderly patients with osteoporosis, or those with a secreting pituitary adenoma under medical treatment (prolactinomas, acromegaly and so on). Patients with thyroid dysfunction (auto-immune, hypo- or hyperthyroidism) benefit from tele-Endo to modify their chronic medical therapy and to ensure a sufficient supply of medication, especially considering the correct titration of anti-thyroid drug, or thyroid treatment during pregnancy, as recently recommended [29].

Tele-Endo resulted a novel measure; however, in our opinion it could be used also after the outbreak, to limit the access to the HCPs. Tele-Endo could be considered to save time in situation of inappropriate health resources, or in view of reducing the geographical distances to the hub, or to save the loss of working hours for employees. In the normal context of everyday clinical practice, a F2F visit is better than a tele-medicine consultation, and the latter can substitute the former only in selected cases, as the renewal of prescriptions for chronic treatment. During tele-medicine visit, physical examinations (as thyroid palpation) is not feasible; moreover, evaluations of anthropometric parameters (weight, height, blood pressure) and data collections for clinical study could be biased by the patient, with possible negative impact on diagnosis, prognosis and clinical studies.

Elderly patients resulted at higher risk for COVID-19 [30]. On the whole, in the 8 weeks of lockdown, 31.8% of those contacted by endocrine triage were ≥ 65 years of age: tele-Endo was applied in more than 60% of their visits. The use of dedicated email, adequate endocrine control and modification of the endocrine treatment during the tele-Endo visit was similar in subgroups of patients, irrespective of age of presentation. The number of re-scheduled or cancelled visits was reduced in geriatric patients after the endocrine-triage. To conclude, our suggested pathway seems confident also for patients ≥ 65 years of age; however, a dedicated study should be considered.

The infective risk is not a minor concern for patients and HWs [31–33], first of all because the patients with an Endocrine conditions could be an asymptomatic carrier [2]. However, all HWs of the AOU of Padova underwent a routine molecular swab for SARS-CoV-2 every 3 weeks, and nowadays none of those working in the Endocrine Outpatient Service is positive. A reduced number of our patients were hospitalized for COVID-19 disease.

Our emergency plan has been developed in few days, and tested in few weeks. Notably, a request for authorization of tele-medicine was submitted from the Italian Society of Endocrinology to the Italian National Institute of Health (Istituto Superiore di Sanità, ISS) on April 29, receiving a positive reply on June 30 [34]. Dedicated platforms and data protection are suggested by the ISS. Since the end of 2019, in Veneto all the medical reports (either from NHS or private health system) as well as the images are reported in the FSEr, a web-based platform dedicated to the storage of medical data (data protection is guaranteed according to the European General Data Protection Regulation– GDPR [35]). Since July, in the AOU of Padova, we can use a dedicated video-platform, where patients can deposit all medical reports in a virtual desktop (available 7 days before the telemedicine visit). High-quality image recorded in the FSEr are available in such platform, patients and physicians can communicate with a camera, and a protected connection with the eCRF of the HCP is used to write the medical reports and send them to the patient.

The endocrine-triage and tele-Endo approaches are not based on systematic reviews or meta-analysis, because there is no evidence on how to adopt endocrine care in times of a health crisis as this outbreak was. Moreover, each plan should be tailored to specific countries, because NHSs could be public, private or mixed. Italy was one of the first severely affected countries in Europe, and Veneto one of those with the higher COVID-19 load, where the containment measures have been applied earlier. During the application of our model, we have noticed some suggestions to improve our clinical practice, not only during an emergency scenario. First, we used phone call for tele-Endo; in the era of connectivity, our Stakeholders should encourage the use of web-based applications that consider patients’ smartphone camera. Moreover, each HCP or region use a web-based system to store data (as FSEr in Veneto); however, they are not connected each other and this represents an issue especially in a hub centre dedicated to rare diseases living in a diffuse geographical area, that performed different biochemical/radiological examinations in different HCPs.

Careful surveillance strategies proposed by Government and HCPs are mandatory to reduce further clusters. We have decided to continue the “stay-safe” procedures, as well as the endocrine triage for selected patients (adrenal insufficiency, hypercortisolism, age ≥ 65 years). We think that our proposed proactive pathway can be really valuable in helping physicians to face circumstances that have not met before.

Our work presents several limitations. First, we do not have a clear research question: we can only propose a description of our routine clinical activity during the outbreak. Therefore, it lacks academic and scientific rigour, because a control group is not feasible.

There is little evidence available on the effectiveness of the Outpatient Service re-organization during a pandemic outbreak. Our strategies were effective in optimizing the spread of the virus (limiting the number of F2F visits) and in continuing the follow-up for patients with chronic endocrine disorders. Our model is far from perfection; however, we managed to give a clinical answer to ≈80% of patients. More time is needed to verify if our model is effective in the long term, but in the short term, we have combined the social distancing with the health of our patients.

## Data Availability

All data analysed during this study are included in this published article or in the data repositories listed in References.

## Abbreviations

AOU: University-Hospital (Azienda Ospedale-Università)
COVID 19: Coronavirus Disease 2019
eCRF: electronic Case Report/Record Form
F2F: Face-to-face
FNA: Fine needle aspiration
GP: General practitioner
HCP: Health-care providers
HW: Health workers
NHS: National Health-care System
SARS-CoV-2: Severe Acute Respiratory Syndrome Coronavirus
US: Ultrasound
WHO: World Health Organization
